# Red yeast rice extract improves lipid metabolism by modulating gut microbiota in high-fat diet mice

**DOI:** 10.3389/fphar.2025.1608582

**Published:** 2025-08-01

**Authors:** Peng Hu, Li Zhang, Hongtao Hu, Di Wang, Jia Chen, Jinghuan Xiao, Hang Wu, Luming Qi, Kaihua Qin, Xiaohong Zuo, Juan Li

**Affiliations:** ^1^ School of Health Preservation and Rehabilitation, Chengdu University of Traditional Chinese Medicine, Chengdu, Sichuan, China; ^2^ School of Comprehensive Health Industry, Sichuan Tourism University, Chengdu, Sichuan, China; ^3^ Department of Spine Surgery, Affiliated Hospital of Shandong Second Medical University, WeiFang, Shandong, China; ^4^ College of Pharmacy, Chengdu University of Traditional Chinese Medicine, Chengdu, Sichuan, China; ^5^ Master of Public Health, College of Veterinary Medicine, Cornell University, Ithaca, NY, United States; ^6^ College of Ophthalmology, Chengdu University of Traditional Chinese Medicine, Chengdu, Sichuan, China

**Keywords:** red yeast rice, high-fat diet, lipid metabolism, gut microbiota, intestinal barrier

## Abstract

As a traditional food-medicine dual-purpose substance, red yeast rice (RYR) has gained wide attention for its lipid-lowering activity. However, existing studies mainly focus on the liver-targeted effects of statin-like components, with limited systematic insights into its lipid metabolism regulation via gut microbiota. This study combines high-fat diet (HFD)-induced hyperlipidemia mouse models, 16S rRNA gene sequencing, untargeted metabolomics, and fecal microbiota transplantation (FMT) to investigate the potential of RYR extract in improving lipid metabolism through gut microbiota modulation. The results showed that RYR extract significantly improved body weight, serum total cholesterol (TC), triglycerides (TG), low-density lipoprotein cholesterol (LDL-C) levels, and hepatic lipid deposition in HFD-fed mice. Additionally, RYR extract effectively restored the intestinal structural damage and enhanced intestinal barrier function. 16S rRNA revealed that RYR extract significantly modulated the gut microbiota, increasing the abundance of beneficial bacteria such as Bifidobacterium and restoring the ratio of Firmicutes to Bacteroidota. Metabolomics analysis revealed that RYR extract significantly modulated the gut microbiota-derived metabolites, particularly in the tryptophan metabolism and phenylalanine metabolism. FMT experiments showed that the fecal microbiota from RYR-treated group obviously improved the blood lipid levels, liver pathology, and intestinal function in HFD-fed mice. These results suggest that RYR extract improves lipid metabolism through the modulation of gut microbiota and related metabolic pathways, which provides new insights into the mechanism research of RYR’s lipid-lowering effect.

## 1 Introduction

In recent years, with changes in lifestyle and the westernization of dietary patterns, lipid metabolism disorders have become a widespread health concern (Wang et al., 2019). These disorders are not only closely associated with chronic diseases such as obesity, diabetes, and hypertension but also serve as one of the major contributors to cardiovascular diseases (Luo et al., 2024; Wang B. et al., 2024). Lipid metabolism disorders caused by an imbalanced diet have emerged as a significant global public health challenge. In particular, high-fat diets (HFDs) not only markedly increase the levels of low-density lipoprotein (LDL) in the blood, leading to atherosclerosis, but also promote visceral fat accumulation and elevate the risk of insulin resistance (Qi et al., 2024a; Wang J. et al., 2024; Yang et al., 2023; Zeng et al., 2023; Kumari et al., 2019). Although several small-molecule drugs are currently available for the prevention and treatment of lipid metabolism disorders, concerns regarding their efficacy and safety remain. Consequently, finding effective strategies to improve lipid metabolism and reduce the incidence of related diseases has become a key focus of medical research. Among various potential interventions, natural products and microbial extracts have garnered extensive attention due to their diverse bioactivities and relatively safe application profiles (Li et al., 2021; 2022; Wang Y.-T. et al., 2024; Wu S. et al., 2023; Zhang et al., 2024; Wang et al., 2025; Wu X. et al., 2023).

Red yeast rice (RYR), a traditional fermented food produced by the fermentation of rice with Monascus spp., has been widely used in Asia as a food seasoning, coloring agent, and traditional medicine (Lee et al., 2023). RYR extracts are rich in bioactive compounds, including monacolin K, red yeast pigments, and gamma-aminobutyric acid, among others (Davani et al., 2024). Early studies have demonstrated that RYR extracts possess multiple biological activities, such as lipid-lowering, antioxidant, anti-inflammatory, and antimicrobial effects (Fukami et al., 2021; Gong et al., 2023; Zhou et al., 2023; Cicero et al., 2019; 2023). However, recent research has suggested that the bioactivities of RYR extracts may not be limited to their direct effects on host metabolic processes but may also involve indirect regulation of lipid metabolism through modulating the composition and function of the gut microbiota.

The gut microbiota, a large and complex microbial ecosystem within the human body, plays a vital role in host nutrient absorption, metabolic regulation, and immune responses. Increasing evidence indicates that gut microbiota dysbiosis is closely related to various metabolic diseases, including obesity, diabetes, and fatty liver disease (Fan and Pedersen, 2021; Qi et al., 2024b). The gut microbiota influences host lipid metabolism through multiple mechanisms, such as regulating intestinal barrier function and affecting amino acid metabolism. Various herbal extracts have been shown to improve host lipid metabolism disorders by modulating the gut microbiota and its metabolites. Studies have suggested that RYR extracts may impact the gut microbiota through multiple pathways (Yang et al., 2021). For instance, monacolin K and red yeast pigments in RYR extracts exhibit antimicrobial activity, selectively inhibiting the growth of harmful bacteria while promoting the proliferation of beneficial microbes (Beltrán et al., 2019; Zhou et al., 2019). However, despite some research exploring the effects of RYR extracts on gut microbiota and their potential metabolic regulatory roles, such studies remain relatively limited.

This study aims to investigate the potential effect that RYR extracts improve lipid metabolism disorders, focusing on their effects on gut microbiota composition, intestinal barrier function, and microbial metabolites. Using animal experiments and multi-omics techniques, we aim to systematically evaluate the intervention effects of RYR extracts on an HFD-induced lipid metabolism disorder model. Additionally, gut microbiota transplantation experiments will be conducted to verify the critical role of gut microbiota in mediating the effects of RYR extracts. These direct evidences of the gut microbiota-lipid metabolism axis provide new insights into the mechanism research of RYR’s lipid-lowering effect.

## 2 Materials and methods

### 2.1 Preparation and components analysis of RYR extract

An appropriate amount of RYR raw material was weighed, ground into fine powder, and passed through an 80-mesh sieve. The RYR powder was then mixed with ultrapure water at a ratio of 6–8 times the weight of the powder and stirred until fully homogenized. The mixture was subjected to ultrasonic extraction at room temperature for 1 h (Xinzhi, Ningbo, China). The extraction residue was collected, mixed with 3–5 times its volume of purified water, and extracted again under identical conditions for 40 min. This process was repeated three times to ensure thorough extraction. The resulting extracts were combined and filtered through a 0.45 µm membrane to remove fine particulates, producing a clear filtrate that was used for subsequent animal intervention experiments.

A high-performance liquid chromatography system (HPLC-2010CHT, Shimadzu, Japan) was employed to determine the lactone components and citrinin content in the RYR extract, ensuring the quality and safety of the RYR. Additionally, an ultra-high-performance liquid chromatography coupled with hybrid quadrupole-orbitrap high-resolution mass spectrometry (UPLC-Q-Orbitrap HRMS) (Thermo Fisher Scientific, Waltham, MA, United States) was used to characterize the metabolomic profile of the RYR extract, providing detailed chemical information on the extract utilized in this study. The detailed methodologies are provided in the Supplementary Material S1.

### 2.2 Animal experiments

C57BL/6 J male mice (6 weeks old, 18–20 g, SPF grade) were obtained from Beijing SiPeiFu Experimental Animal Co., Ltd. (Beijing, China). After an acclimatization period of 7 days, the mice were randomly allocated into four experimental groups: the normal diet group (ND, n = 6), the high-fat diet group (HFD, n = 6), the low-dose RYR extract group (RYR_L, 200 mg/kg/day, 1.8 mg/kg/day monacolin K, n = 6), and the high-dose RYR extract group (RYR_H, 400 mg/kg/day, 3.6 mg/kg/day monacolin K, n = 6). In the RYR_L and RYR_H groups, mice were administered different concentrations of RYR extract by gavage while continuously fed an HFD; the ND and HFD groups were gavaged with an equivalent volume of physiological saline and fed a normal diet or HFD, respectively. During the experiment, mice were housed in a barrier environment with a temperature of (22 ± 2)°C, humidity of 50% ± 10%, and a 12-h light/dark cycle, with free access to food and water.

After the 6-week dietary intervention, 6 mice were selected from each group for sample collection. Blood was collected via retro-orbital venous plexus puncture into EDTA-coated tubes. The tubes were centrifuged at 3,000 × g for 15 min at 4°C to separate the serum. The supernatant was aliquoted into cryotubes and stored at −80°C.

Prior to euthanasia, fresh fecal pellets were collected by gently pressing the lower abdomen and stimulating the anus to induce defecation. Sterile forceps were used to collect 3–5 fecal pellets from each of the 6 mice per group. Samples were immediately snap-frozen in liquid nitrogen and stored at −80°C for microbiome and metabolomics analysis. After laparotomy, the entire liver was excised, rinsed with ice-cold PBS, and blotted dry. Approximately 0.5 g of tissue from the left liver lobe was fixed in 4% paraformaldehyde for histological staining, while the remaining liver tissue was snap-frozen in liquid nitrogen and stored at −80°C for biochemical analysis. The colon was carefully dissected, flushed with ice-cold PBS to remove luminal contents, and divided into two parts: the proximal segment (2 cm) was fixed in 4% paraformaldehyde for morphological evaluation, and the distal segment was snap-frozen in liquid nitrogen for biochemical analysis. All experimental protocols were reviewed and approved by the Experimental Animal Welfare and Ethics Committee of Chengdu University of Traditional Chinese Medicine (Approval No. 2020-35), adhering to international ethical standards and guidelines.

### 2.3 Biochemical analysis

Blood samples were drawn from the mice via the retro-orbital sinus and transferred into EP tubes preloaded with sodium heparin. The samples were centrifuged at 3,500 rpm for 15 min to separate the plasma. The concentrations of triglycerides (TG), total cholesterol (TC), high-density lipoprotein cholesterol (HDL-C), and low-density lipoprotein cholesterol (LDL-C) in plasma, as well as TG and TC levels in liver tissues, were quantified using a fully automated biochemical analyzer (Mindray, BS-200) along with commercially available kits (Mindray, Shenzhen, China). Plasma levels of adiponectin and leptin were assessed using dedicated assay kits (Elabscience, Wuhan, China).

### 2.4 Histopathological analysis

Liver and colon tissues were excised from the mice and preserved in 4% paraformaldehyde. Following fixation, the samples were subjected to an ethanol gradient for dehydration, cleared, embedded in paraffin, sectioned, and deparaffinized prior to staining. Histological analysis of the liver and colon was performed using Hematoxylin and Eosin (HE) staining to assess structural and morphological characteristics. Fresh liver tissue, after snap-freezing, was sectioned using a cryostat, fixed in 4% paraformaldehyde, and stained with Oil Red O (ORO) to evaluate lipid deposition.

### 2.5 Immunofluorescence and PCR analysis

The collected tissues were fixed in 4% paraformaldehyde for at least 24 h. Subsequently, the samples underwent deparaffinization, rehydration, antigen retrieval, and blocking steps. For immunofluorescence analysis of colon tissues, primary antibodies (ZO-1, occludin, claudin-1) were diluted and incubated overnight at 4°C. The sections were then treated with FITC-conjugated secondary antibodies and counterstained with DAPI. After staining, the slides were mounted with antifade mounting medium and observed under a fluorescence microscope.

Total RNA was extracted from colon tissues, and cDNA was synthesized using a reverse transcription kit (Chengdu Rongwei Gene Biotechnology Co., Ltd., Sichuan, China). The synthesized cDNA was used as a template for real-time quantitative PCR (qPCR) (Hangzhou Bioer Technology Co., Ltd., Zhejiang, China) to determine the relative expression levels of ZO-1, occludin, and claudin-1. The qPCR reactions were set up according to the manufacturer’s instructions, with the following conditions: initial denaturation at 95°C for 2 min, followed by 40 cycles of 95°C for 15 s, 60°C for 30 s, and 72°C for 30 s β-actin was used as the reference gene, and the relative expression levels of target genes were calculated using the 2^(-ΔΔCt) method.

### 2.6 16S rRNA sequencing and analysis

Genomic DNA was isolated from fecal samples using a stool DNA extraction kit (Cwbio, Jiangsu, China). The quality and concentration of the extracted DNA were assessed using agarose gel electrophoresis and a NanoDrop^®^ ND-2000 spectrophotometer (Thermo Scientific Inc., United States). The V3-V4 region of the 16S rRNA gene was amplified using universal primers, and the PCR products were purified, quantified, and sequenced on the Illumina NovaSeq 6000 platform. Data analysis was performed using the QIIME2 pipeline, where amplicon sequence variants (ASVs) were identified with DADA2, and taxonomic classification was conducted using the VSEARCH algorithm against the Silva database.

α-diversity metrics, such as the Shannon index and Chao1 index, were used to evaluate species richness and diversity within individual samples. β-diversity was employed to compare microbial community composition between samples, with visualizations generated through principal coordinates analysis (PCoA) and non-metric multidimensional scaling (NMDS) (Qi et al., 2024a). Differential abundance analysis was performed using methods like linear discriminant analysis effect size (LEfSe) and volcano plots to identify significantly altered microbial taxa between experimental groups or conditions.

### 2.7 Gut metabolomics detection and analysis

To analyze the gut metabolome, 200 mg of fecal samples were collected. Each sample was finely ground and subjected to ultrasonic extraction in an ice water bath for 10 min. The resulting mixture was centrifuged at 13,000 rpm for 10 min, and 100 μL of the supernatant was collected. This was mixed with 200 μL of acetonitrile, vortexed for 60 s to precipitate proteins, and centrifuged again at 13,000 rpm for another 10 min. The supernatant was transferred to 1.5 mL EP tubes and vacuum-dried. The dried residues were reconstituted in 200 μL of extraction solution (acetonitrile:water = 4:1, v/v), vortexed for 60 s, and centrifuged at 13,000 rpm at 4°C for 10 min. The final supernatants were filtered through a 0.22 μm membrane and prepared for metabolomic analysis. The metabolomic profiling was performed using UPLC-QE-Orbitrap-MS, with detailed mass spectrometry parameters provided in Supplementary Material S2.

### 2.8 FMT experiment

Healthy C57BL/6 mice, aged 6–8 weeks, were selected and treated with a broad-spectrum antibiotic mixture via drinking water for 7 consecutive days to construct pseudo sterile (PS) mice. The antibiotic solution consisted of ampicillin (1 g/L), neomycin (1 g/L), metronidazole (1 g/L), and vancomycin (0.5 g/L)(Lu et al., 2019). The solution was refreshed daily to ensure the antibiotics remained effective. The mice were then randomly divided into two groups. The control group was fed a HFD and gavaged daily with an equal volume of distilled water, while the intervention group was also fed a HFD but gavaged daily with a high dose of fecal extract derived from mice in RYR_H group. After 4 weeks of intervention, blood samples were collected to measure lipid profile parameters (TG, TC, HDL-C, LDL-C) as well as leptin and adiponectin levels. Liver and colon tissues were collected to prepare histological sections for the observation of tissue changes, clarifying the effects of RYR intervention on lipid metabolism and its association with gut microbiota.

### 2.9 Data analysis

Numerical data are presented as mean ± standard deviation and derived from at least three independent experiments. Statistical analyses were performed using GraphPad Prism 9.0 software to evaluate the significance of intergroup differences. First, normality tests were conducted on the data. If the data conformed to a normal distribution, one-way analysis of variance (One-way ANOVA) was used for intergroup comparisons, followed by Tukey’s *post hoc* test for multiple comparisons. If the data did not conform to a normal distribution, nonparametric tests were applied, with Dunn’s *post hoc* test used to analyze intergroup differences. For multivariate analyses, an adjusted p-value <0.05 was considered statistically significant. Significant differences among the ND group, HFD group, and intervention groups were indicated by asterisks: * for p < 0.05, ** for p < 0.01, and *** for p < 0.001.

## 3 Results

### 3.1 Chemical composition analysis of RYR extract

RYR extract is a complex mixture of multiple components, and its biological activity results from the combined effects of various metabolites. To better understand the primary chemical basis for the lipid-lowering effects of RYR, we first conducted both qualitative and quantitative analyses of its chemical composition.

**FIGURE 1:**
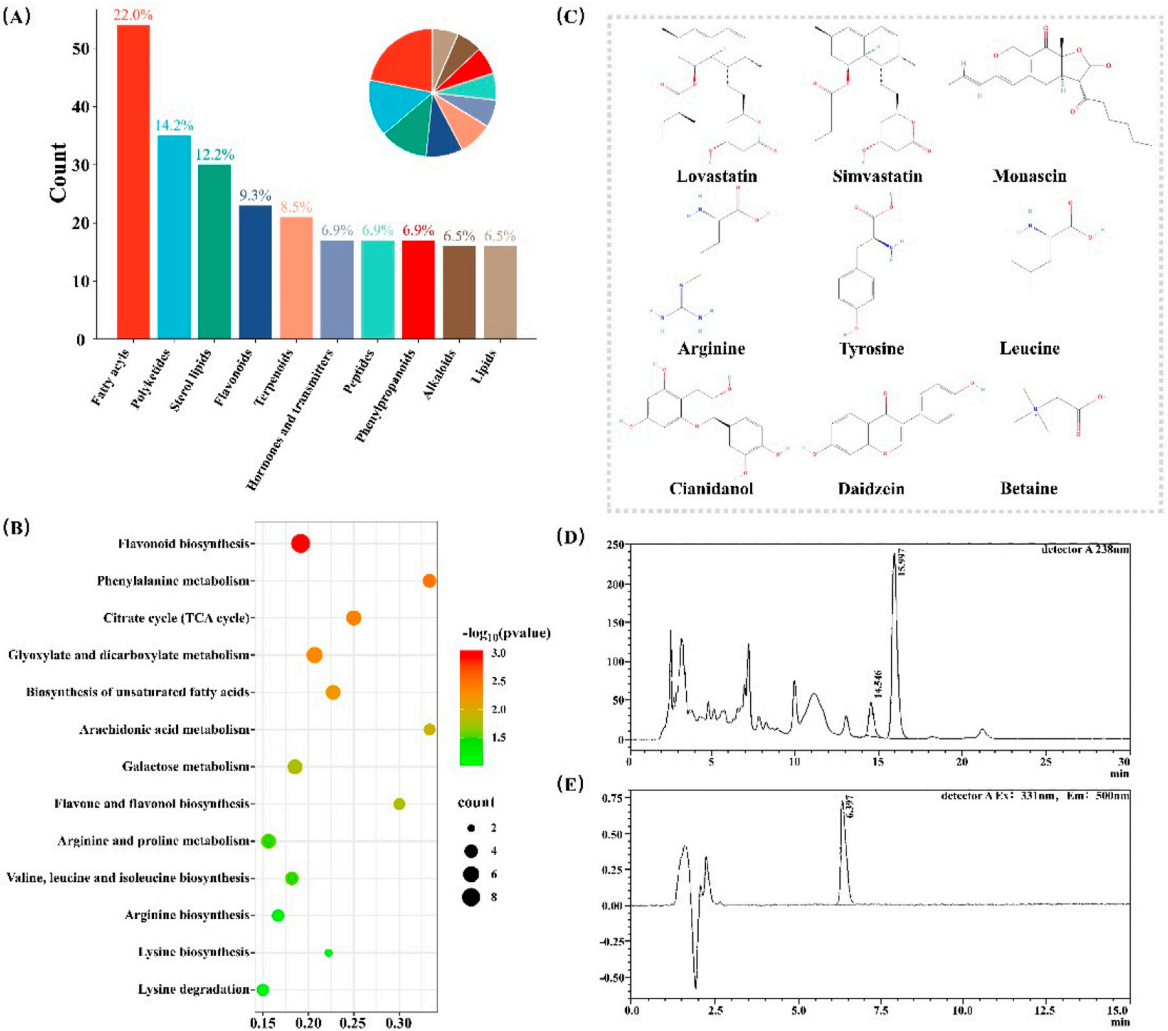
Chemical Composition Analysis of RYR Extract. **(A)** The classification of annotated compounds; **(B)** KEGG analysis of the annotated compounds; **(C)** Nine compounds identified based on standard substances; **(D)** Chromatogram of Monacolin K quantitative analysis; **(E)** Chromatogram of citrinin quantitative analysis.

We initially characterized the chemical profile of the RYR extract using UPLC-Q-Orbitrap HRMS technology. we performed a comparison using the mzCloud database and identified 539 metabolites that were annotated in the KEGG database (Supplementary Material S3). These metabolites predominantly belong to categories such as Fatty Acyls, Polyketides, Sterol Lipids, Flavonoids, and Terpenoids (Figure 1A). KEGG pathway analysis indicated that these metabolites are primarily involved in flavonoid biosynthesis and phenylalanine metabolism (Figure 1B).

Subsequently, based on experimental chemical standards and tandem mass spectrometry identification, a total of nine distinct compounds were identified in RYR extract (Figure 1C). These compounds include Monacolin K, Simvastatin, Monascin, Arginine, Tyrosine, Leucine, Catechin, Daidzein, and Betaine (Supplementary Material S4).

More, we quantitatively measured the levels of these two components (Monacolin K and citrinin) in the extract, which are two major quality control components in RYR (Fogacci et al., 2019). The results showed that the content of Monacolin K in the RYR extract was 0.9% (Figure 1D), while the content of Simvastatin was 34 μg per kilogram (Figure 1E). These data not only confirm the chemical diversity of the RYR extract but also ensure its efficacy and safety.

### 3.2 Effects of RYR extract on lipid metabolism in HFD mice

**FIGURE 2:**
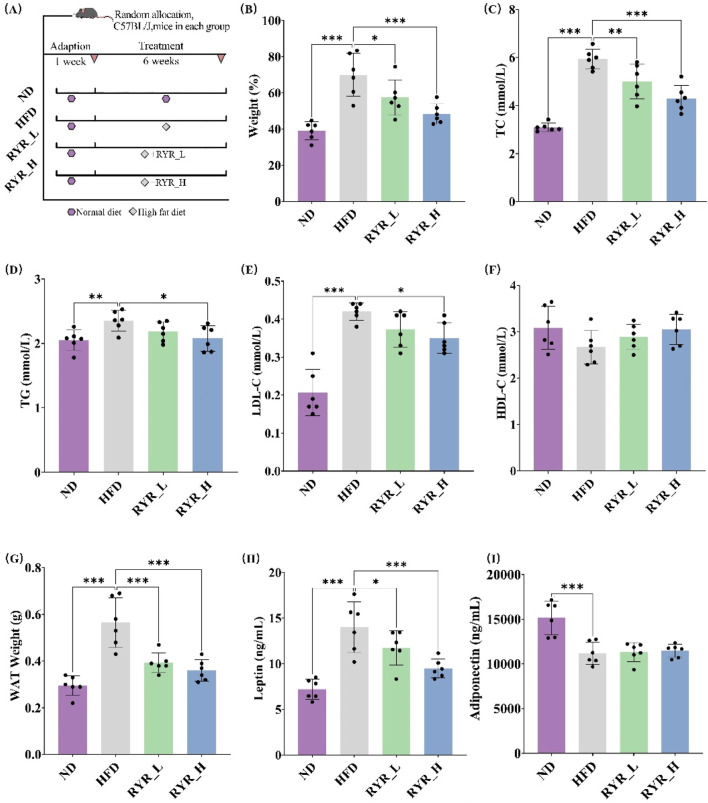
Effects of RYR extract on lipid metabolism in HFD mice. **(A)** Procedure of animal experiments; **(B)** Comparison analysis of body weight; **(C)** Comparison analysis of TC; **(D)** Comparison analysis of TG; **(E)** Comparison analysis of LDL-C; **(F)** Comparison analysis of HDL-C; **(G)** Comparison analysis of WAT weight; **(H)** Comparison analysis of leptin; **(I)** Comparison analysis of adiponectin. (Note: * for p < 0.05, ** for p < 0.01, and *** for p < 0.001).

To investigate the effects of RYR extract on lipid metabolism, we established a mouse model of lipid metabolism disorder induced by a 6-week HFD (Figure 2A). The intervention effects were evaluated by assessing the impacts of HFD and different doses of RYR extract on body weight, serum lipid levels, WAT weight, and adipokines, including leptin and adiponectin.

The results showed that HFD significantly increased body weight (p < 0.001), whereas interventions with RYR_L (p < 0.05) and RYR_H (p < 0.001) significantly alleviated HFD-induced weight gain (Figure 2B). Additionally, HFD markedly altered plasma lipid levels, significantly increasing TC (p < 0.001), TG (p < 0.01), and LDL-C (p < 0.001). Treatment with RYR at both doses, particularly RYR_H, significantly reduced TC (p < 0.001), TG (p < 0.05), and LDL-C (p < 0.05) levels (Figures 2C–E). Regarding HDL-C levels, neither HFD nor RYR interventions induced significant changes (Figure 2F), suggesting that the lipid-lowering effects of RYR extract are primarily focused on reducing harmful lipids.

Adipokines are important indicators that directly reflect changes in lipid metabolism and are crucial for evaluating the effects of RYR extract on lipid metabolism. The study results revealed that both doses of RYR extract significantly reversed the HFD-induced increase in WAT weight (p < 0.001) (Figure 2G). A 6-week HFD significantly elevated leptin levels in mice (p < 0.001), while both RYR_L (p < 0.05) and RYR_H (p < 0.001) interventions significantly reduced leptin levels, with RYR_H showing a more pronounced effect (Figure 2H). Although HFD markedly reduced adiponectin levels (p < 0.001), RYR treatment did not exhibit a regulatory effect on adiponectin (Figure 2I). These findings suggest that RYR has a certain regulatory effect on adipose tissue function and hormone secretion.

In conclusion, RYR extract significantly ameliorates HFD-induced metabolic disorders in mice, including alleviating weight gain, improving lipid profiles, and modulating levels of relevant adipokines.

### 3.3 Effect of RYR extract on liver lipid metabolism in HFD mice

**FIGURE 3:**
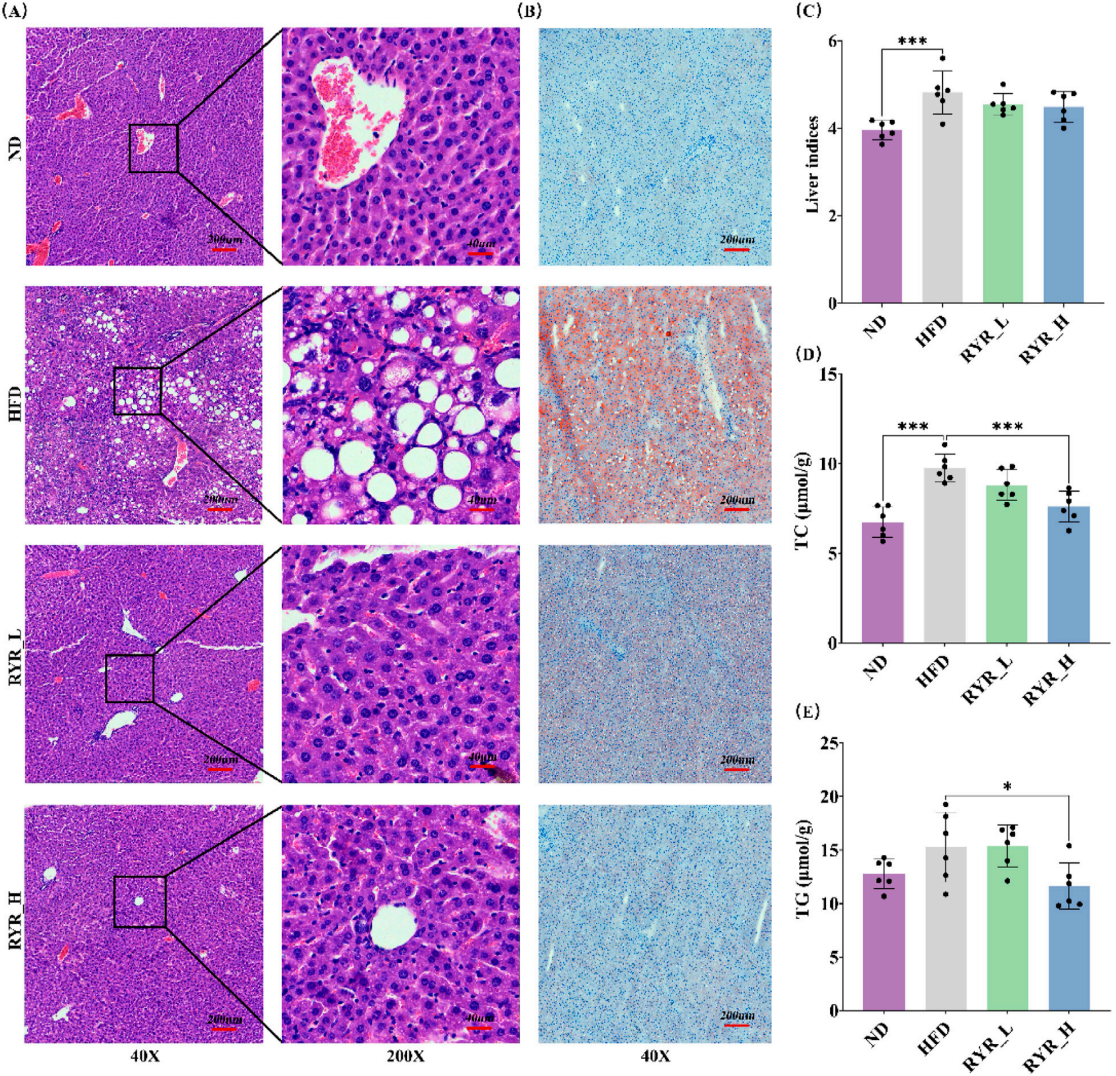
Effect of RYR extract on liver lipid metabolism in HFD mice. **(A)** HE staining of liver tissue sections; **(B)** ORO staining of liver tissue sections; **(C)** Comparison analysis of liver index; **(D)** Comparison analysis of TC; **(E)** Comparison analysis of TG. (Note: * for p < 0.05, ** for p < 0.01, and *** for p < 0.001).

HE staining results showed that HFD induced the formation of numerous lipid vacuoles in the liver, indicating hepatic steatosis. Intervention with RYR at both doses significantly reduced the number of lipid vacuoles, with a more pronounced improvement observed in the RYR_H group (Figure 3A).

Similarly, ORO staining revealed significant lipid deposition in the livers of HFD-fed mice, as evidenced by an increase in positive staining areas. Both doses of RYR extract significantly reduced lipid deposition, with the RYR_H group demonstrating superior efficacy (Figure 3B).

In addition, we evaluated the effects of RYR extract on the liver index as well as hepatic TC and TG levels in HFD-fed mice. The results showed that HFD significantly increased the liver index (p < 0.001), while RYR extract at both doses slightly reduced this parameter, suggesting that RYR extract may improve liver function by alleviating hepatic steatosis (Figure 3C). Furthermore, a 6-week HFD significantly increased hepatic TC levels (p < 0.001) and mildly elevated TG levels. High-dose RYR extract significantly reduced TC (p < 0.001) and TG (p < 0.05) levels (Figures 3D,E), confirming that the RYR_H group exhibited more pronounced improvements in hepatic lipid metabolism and structural damage.

In summary, liver histopathological observations and related indicators demonstrate that RYR extract significantly ameliorates HFD-induced hepatic steatosis and hepatic lipid metabolism abnormalities.

### 3.4 Effect of RYR extract on intestinal function in HFD mice

**FIGURE 4:**
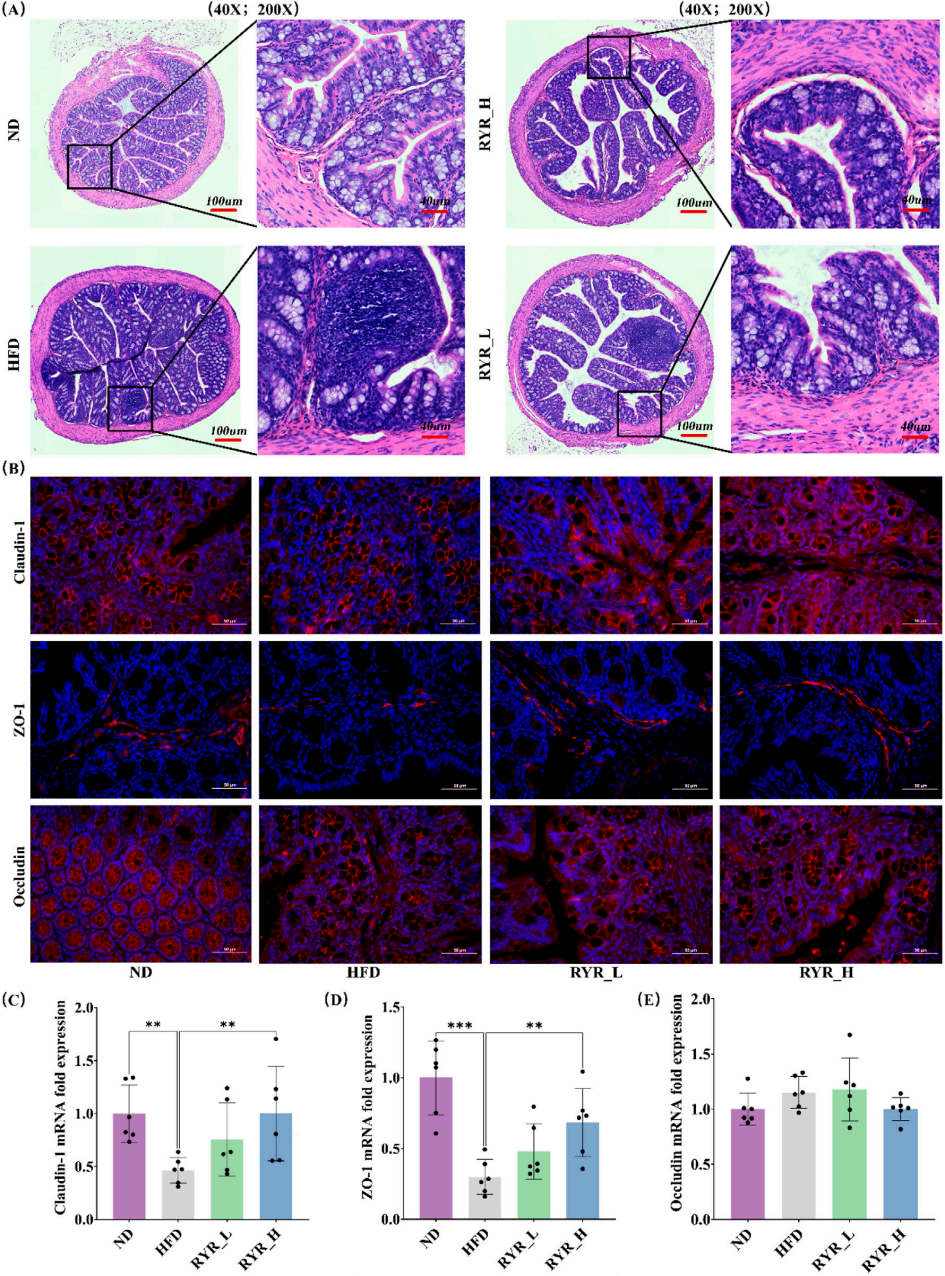
Effect of RYR extract on intestinal function in HFD mice. **(A)** HE staining of colon tissue sections; **(B)** Changes in Claudin-1, ZO-1, and Occludin immunofluorescence; **(C)** Relative expression of Claudin-1 gene; **(D)** Relative expression of ZO-1 gene; **(E)** Relative expression of Occludin gene. (Note: * for p < 0.05, ** for p < 0.01, and *** for p < 0.001).

We investigated the effects of RYR extract on intestinal function in HFD-fed mice. HE staining results showed that the intestinal structure in the ND group was normal, with intact mucosa and well-defined villi. In contrast, HFD-fed mice exhibited shortened and detached villi, disrupted cellular structure in the intestinal lumen, and impaired intestinal barrier function. Both RYR_L and RYR_H interventions significantly alleviated the HFD-induced intestinal damage, with RYR_H showing the most pronounced effects. The intestinal villi in the RYR_H group were notably restored, with structural features comparable to those in the ND group (Figure 4A).

Immunofluorescence staining revealed that HFD significantly downregulated the protein expression levels of Claudin-1 and ZO-1. Both RYR_L and RYR_H interventions restored their expression levels, with RYR_H achieving the most substantial recovery. No significant changes in Occludin protein expression levels were observed between the HFD and RYR-treated groups (Figure 4B). Additionally, qPCR analysis of the relative mRNA expression levels of these tight junction proteins showed results consistent with those of the immunofluorescence analysis (Figures 4C–E). Based on histological observations and molecular analyses, RYR extract significantly improved HFD-induced intestinal barrier dysfunction. These findings suggest that RYR may enhance intestinal barrier function, thereby mitigating systemic lipid metabolism disorders.

### 3.5 Effect of RYR extract on gut microbiota composition in HFD mice

**FIGURE 5:**
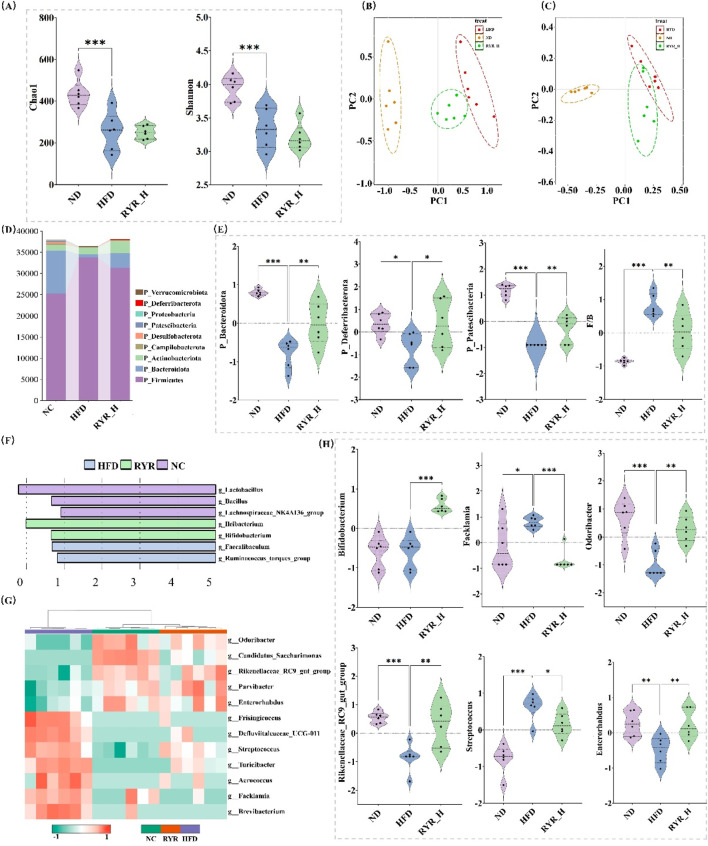
Effect of RYR extract on gut microbiota composition in HFD mice. **(A)** Comparison of gut microbiota diversity; **(B)** PCoA analysis of different groups; **(C)** NMDS analysis of different groups; **(D)** Changes in phylum-level gut bacterial proportions; **(E)** Phylum-level changes in gut bacterial abundance; **(F)** Lefse analysis; **(G)** Cluster heatmap analysis; **(H)** Comparison analysis of key bacterial genera. (Note: * for p < 0.05, ** for p < 0.01, and *** for p < 0.001).

To investigate the effects of HFD-induced gut dysbiosis, we analyzed the microbial diversity and composition in RYR_H-treated mice, as the high-dose group showed the best intervention effects. The results of α-diversity analysis revealed that HFD significantly reduced Chao1 index (p < 0.001) and Shannon index (p < 0.001), indicating that HFD caused a marked decline in gut microbiota diversity and richness. However, interventions with RYR did not significantly reverse these changes (Figure 5A).

To assess the impact of RYR extract on gut microbial β-diversity, we performed PCoA and NMDS. The results demonstrated that the gut microbiota composition of the HFD group was significantly separated from that of the ND group, indicating profound alterations induced by HFD. Notably, the microbial composition in the RYR_H group shifted closer to that of the ND group, further supporting the role of RYR_H in mitigating HFD-induced microbial dysbiosis (Figures 5B,C).

At the phylum level, both HFD and high-dose RYR intervention significantly altered the relative abundance of various bacterial phyla (Figure 5D). Notably, HFD markedly reduced the abundance of Bacteroidota, Deferribacterota, and Patescibacteria, while high-dose RYR intervention significantly increased their abundance (p < 0.01, p < 0.05, p < 0.01, respectively). Moreover, RYR_H treatment notably restored the Firmicutes/Bacteroidota ratio (p < 0.001), indicating that high-dose RYR extract significantly mitigated HFD-induced gut microbial dysbiosis (Figure 5E).

To further investigate the effects of HFD and high-dose RYR intervention on gut microbiota at the genus level, we performed Lefse analysis and hierarchical clustering via heatmap. The results revealed that *Lactobacillus* was enriched in the NC group, while high-dose RYR intervention significantly increased the abundance of Heibacterium and Bifidobacterium (Figure 5F). Additionally, Figure 5G shows that high-dose RYR intervention markedly reversed HFD-induced changes in the abundance of several microbial genera. Some of these significantly altered genera were visualized and are highlighted as candidates for further investigation (Figure 5H).

In summary, RYR extract significantly improved HFD-induced gut dysbiosis and restored gut microbial balance. These findings further support the potential application of RYR in the prevention and treatment of metabolic diseases, particularly through the regulation of gut microbiota to enhance systemic metabolic health.

### 3.6 Effects of RYR extract on gut microbiota-derived metabolites in HFD mice

To investigate this, we employed UPLC-QE-Orbitrap-MS to analyze the effects of HFD treatment and RYR_H extract on gut metabolites in mice. Following data cleaning and preprocessing, metabolites with an RSD of less than 30% were retained for further analysis.

**FIGURE 6:**
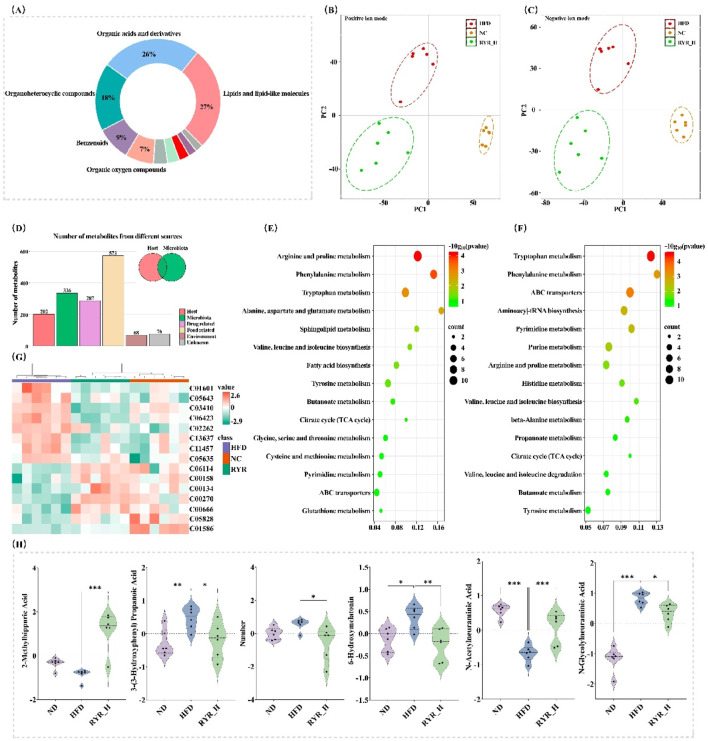
Effects of RYR extract on gut microbiota-derived metabolites in HFD mice. **(A)** Classification of identified metabolites; **(B)** PLS-DA analysis in positive ion mode; **(C)** PLS-DA analysis in negative ion mode; **(D)** Statistical analysis of metabolites from different sources; **(E)** KEGG analysis of differential metabolites between NC and HFD groups; **(F)** KEGG analysis of differential metabolites between HFD and RYR_H groups; **(G)** Cluster heatmap analysis; **(H)** Comparison analysis of important differential metabolites. (Note: * for p < 0.05, ** for p < 0.01, and *** for p < 0.001).

These metabolites were classified into five major categories: organic acids and derivatives (27%), lipids and lipid-like molecules (27%), organic heterocyclic compounds (18%), benzenoids (9%), and organic oxygen compounds (7%) (Figure 6A). These results indicate that gut microbiota-derived metabolites are highly diverse, encompassing key molecules involved in energy metabolism, lipid metabolism, and amino acid metabolism.

PLS-DA analysis was conducted to evaluate the overall changes in metabolite composition among the experimental groups. The results demonstrated a clear separation among the NC, HFD, and RYR_H groups under both positive and negative ion modes, indicating that both HFD treatment and RYR_H intervention had significant and broad impacts on gut metabolites in mice (Figures 6B,C).

Subsequently, we used the “MetOrigin” tool to trace these metabolites and identified a total of 336 metabolites associated with the gut microbiota. Among them, 158 metabolites were derived from microbial metabolism, and 178 were products of co-metabolism between microbes and the host (Figure 6D). Volcano plot analysis of these gut microbiota-derived metabolites revealed that HFD significantly downregulated 68 metabolites (p < 0.05) and significantly upregulated 65 metabolites (p < 0.05) (Supplementary Material S5). These altered metabolites were primarily enriched in the “Arginine and proline metabolism”, “Phenylalanine metabolism”, and “Tryptophan metabolism” pathways (Figure 6E).

Following high-dose RYR intervention, 47 metabolites were significantly downregulated (p < 0.05), and 72 metabolites were significantly upregulated (p < 0.05) (Supplementary Material S6). The significantly altered metabolites were mainly enriched in the “Tryptophan metabolism”, “Phenylalanine metabolism”, and “ABC transporters” pathways (Figure 6F). As shown in Figures 6G, 15 metabolites were significantly reversed after high-dose RYR treatment. We visualized several key metabolites significantly reversed in the tryptophan metabolism and phenylalanine metabolism pathways, which warrant further investigation (Figure 6H).

In summary, high-dose RYR extract significantly modulated the HFD-induced alterations in gut microbiota-derived metabolites. The tryptophan metabolism and phenylalanine metabolism pathways may represent potential mechanisms by which RYR influences lipid metabolism.

### 3.7 Effects of FMT experiment on lipid metabolism and tissue pathology in HFD mice

**FIGURE 7:**
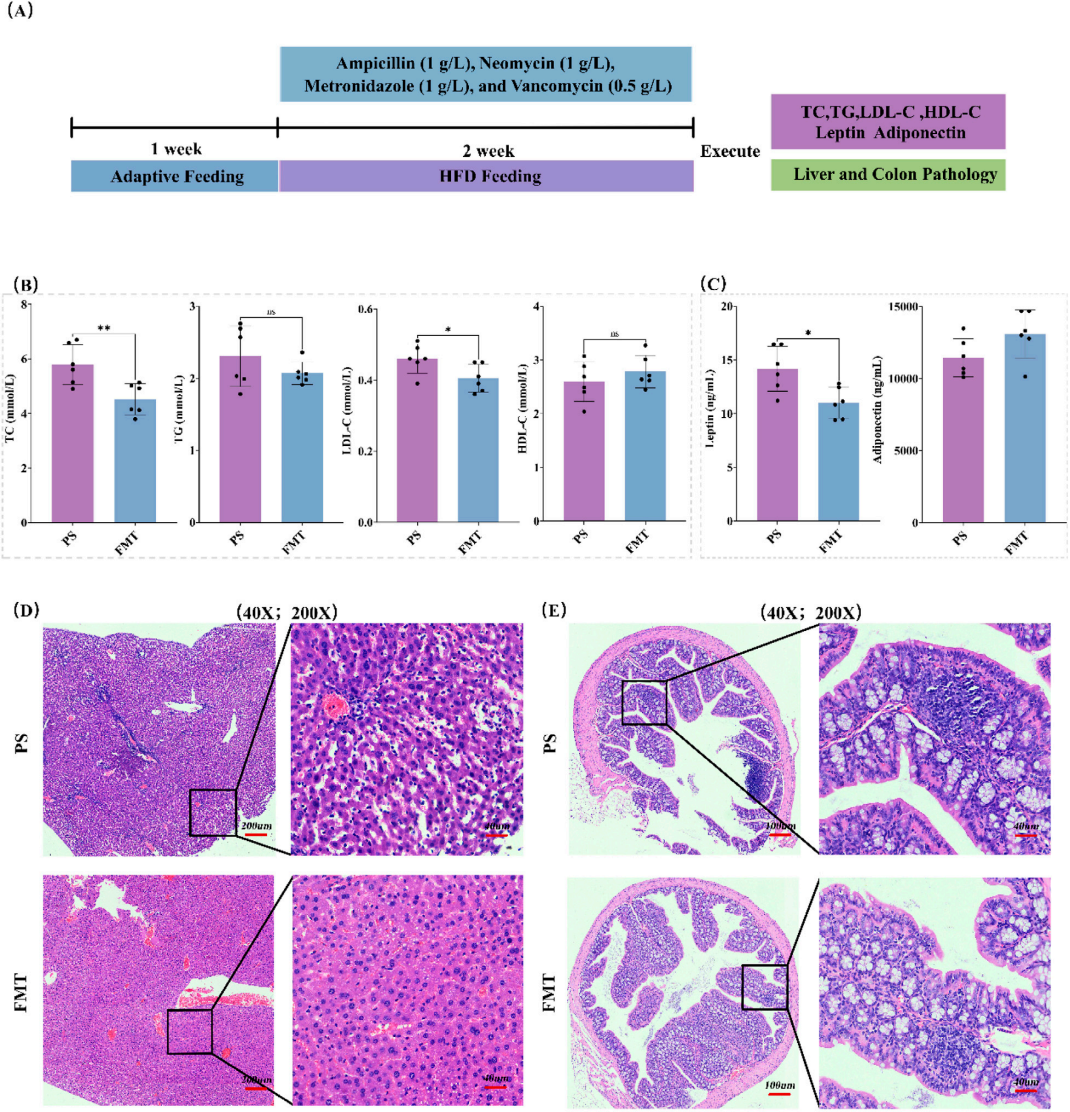
Effects of FMT experiment on lipid metabolism and tissue pathology in HFD mice. **(A)** FMT experimental procedure; **(B)** Changes in blood lipid levels in the FMT experiment; **(C)** Changes in leptin and adiponectin levels in the FMT experiment; **(D)** HE staining analysis of liver tissue; **(E)** HE staining analysis of colon tissue. (Note: * for p < 0.05, ** for p < 0.01, and *** for p < 0.001).

To investigate whether RYR extract improves lipid metabolism in HFD mice through modulating the gut microbiota, we constructed an HFD-induced PS mouse model using a four-antibiotic cocktail (Figure 7A). The results showed that mice in the RYR_H group exhibited significant changes in the levels of TC (p < 0.01) and LDL-C (p < 0.05) in fecal extracts compared to HFD mice (Figure 7B). Additionally, the leptin levels were significantly reduced (p < 0.05) in the FMT group, while adiponectin levels obviously increased (Figure 7C).

Histological analysis of liver tissue using H&E staining revealed that the PS group showed fatty degeneration of hepatocytes and mild inflammatory infiltration, while the FMT group exhibited significant improvement in liver histopathology, with more regular hepatocyte arrangement and reduced inflammation (Figure 7D). In the colon, the PS group exhibited a large number of lymphocytes in intestinal mucosal layer and the submucosal stroma, with neutrophil infiltration observed in some areas, whereas the FMT group showed more intact colon structure, with goblet cells restored to normal distribution and a significant improvement in inflammation (Figure 7E).

These findings suggest that FMT partially improves lipid metabolism dysfunction and liver-intestinal tissue pathology in HFD-induced mice, indicating that red yeast rice extract improves lipid metabolism in HFD mice, at least in part, by modulating the gut microbiota.

## 4 Discussions

RYR, as a traditional Chinese medicine, has been used for centuries to regulate lipid metabolism. This study found that RYR extract significantly reduced serum TC, TG, and LDL-C levels in HFD-fed mice, demonstrating a strong lipid metabolism-regulating effect. Similar studies have also shown that RYR extract plays a role in regulating blood lipids, with Monacolin K being the main active ingredient widely recognized for its significant effect on lowering blood lipids (Huang et al., 2013; Mazza et al., 2018). However, the chemical composition analysis based on UPLC-Q-Orbitrap HRMS revealed that, in addition to Monacolin K, RYR extract also contains various other chemical components such as pigments and polypeptides (Srianta et al., 2016), which have also been shown to have significant lipid-lowering effects. These small molecular metabolites are worth further investigation.

The liver is the central organ of lipid metabolism (Ramatchandirin et al., 2023), and studying the alleviation of hepatic lipid accumulation can reveal the protective effects of RYR extract against lipid metabolism disorders. RYR extract significantly alleviated hepatic fat accumulation and structural damage in HFD-fed mice (Banach et al., 2022). This finding is consistent with previous studies, indicating that RYR extract can improve hepatic fat degeneration and protect liver function (Kwon et al., 2023). Further biochemical analysis revealed that the RYR_H group significantly reduced liver TC and TG levels, supporting the potential application of RYR in regulating hepatic lipid metabolism.

Intestinal function plays a crucial role in lipid metabolism (Hu et al., 2021; Song et al., 2023) and is a key factor in maintaining metabolic balance. Our study shows that RYR extract can improve the intestinal structural damage induced by HFD, restoring the morphology of intestinal epithelial cells and increasing the expression of tight junction proteins such as Claudin-1 and ZO-1. These results suggest that RYR extract may enhance intestinal barrier function (Qi et al., 2024b; Wu et al., 2022), preventing the entry of metabolic-related endotoxins and inflammatory factors into circulation, thereby improving systemic lipid metabolism abnormalities.

The gut microbiota plays a crucial role in regulating host lipid metabolism, and alterations in its composition and function are closely associated with metabolic disorders (Brown et al., 2023). We found that RYR extract significantly restored the HFD-induced imbalance in the gut microbiota of mice, especially increasing the abundance of beneficial bacteria such as Bifidobacterium and Heibacterium, while significantly restoring the ratio of Firmicutes to Bacteroidota. This is consistent with several studies indicating that gut microbiota imbalance may be a major factor in metabolic disorders (Fan and Pedersen, 2021; Wu et al., 2021). Gut-derived metabolites serve as key mediators by which the gut microbiota influences lipid metabolism (Jung et al., 2024). RYR extract, by improving gut microbiota composition, may indirectly regulate tryptophan metabolism and phenylalanine metabolism pathways, as well as the production of gut-derived metabolites, thereby helping to restore lipid metabolism balance.

FMT experiments can directly demonstrate the significant role of the gut microbiota in regulating host lipid metabolism (Zikou et al., 2024), thereby providing evidence for the relationship between gut microbiota and human health. In the FMT experiment, we found that RYR extract, by modulating the gut microbiota, partially improved lipid metabolism disorders and liver tissue pathology in HFD-fed mice. This result suggests that the metabolic improvement effect of RYR extract partly depends on the modulation of the gut microbiota. This finding supports the role of gut microbiota as a critical mediator in RYR’s regulation of metabolism and further suggests the potential application of RYR as a prebiotic or gut microbiota modulator in metabolic diseases.

Although this study reveals the potential of RYR extract to improve lipid metabolism by regulating gut microbiota, several limitations warrant attention. Mechanistically, the current associations between differentially abundant microbes and metabolites are based solely on correlational analyses, lacking causal validation experiments such as germ-free animal colonization or single-strain co-culture assays. Additionally, whether RYR mediates metabolic improvements through specific microbial-derived signaling molecules or host receptors (e.g., FXR, PPARγ) remains unclear, requiring further exploration using targeted metabolomics and molecular biology techniques. In terms of experimental models, the pathological complexity of the animal model used differs from that of human metabolic diseases, necessitating caution in extrapolating findings to clinical settings. Moreover, the study’s short experimental duration lacks long-term toxicity data, and the dose-dependent risks of natural components in RYR extract were not systematically monitored. From a material basis perspective, the chemical characterization of red yeast rice is incomplete—for example, quantitative analysis of key active components (e.g., total monacolin K content, homolog composition such as monacolin J/L derivatives, and other functional substances) remains insufficient to clarify its primary active ingredients and potential synergistic mechanisms. Future research should prioritize causal mechanism validation, construction of cross-organ regulatory networks, long-term toxicity evaluation, small-sample clinical cohort studies, and comprehensive chemical characterization to systematically dissect the mechanisms of action, assess safety, clarify the material basis, and evaluate clinical translation potential.

## 5 Conclusion

This study reveals the potential of RYR extract in improving lipid metabolism by regulating the gut microbiota. Our results indicate that RYR extract not only significantly improves body weight, blood lipid levels, and hepatic lipid accumulation induced by a high-fat diet in mice, but also effectively restores intestinal barrier function. Furthermore, RYR extract promotes systemic lipid metabolism health by modulating the composition of the gut microbiota. This finding provides new theoretical evidence for the application of RYR extract as a prebiotic or microbiota modulator in metabolic diseases.

## Data Availability

The 16S rRNA sequencing data presented in the study are deposited in Figshare, available at https://doi.org/10.6084/m9.figshare.29545820.v1.
