# Supplementary material for: Red yeast rice extract improves lipid metabolism by modulating gut microbiota in high-fat diet mice
Source: Front Pharmacol. 2025 Aug 1;16:1608582. doi: 10.3389/fphar.2025.1608582 (PMC12353709; doi:10.3389/fphar.2025.1608582)
Supplement: Supplementary file 1 [file Supplementaryfile1.docx]

**Supplementary material 1: Components analysis of RYR extract**

The Shimadzu High-Performance Liquid Chromatograph (LC-2010CHT) was used to determine the characteristic chemical components in the red yeast rice extract. Accurately weigh 0.543g of red yeast rice raw material into a 50mL volumetric flask, add 30mL of 75% ethanol, and sonicate at room temperature for 50 minutes. Centrifuge at 3500 r/min for 10 minutes, then filter the supernatant through a 0.45 µm micropore filter. The chromatographic conditions were as follows: flow rate 1.0 mL/min; column temperature 25°C; injection volume 20 µL; detection wavelength 238 nm; chromatographic column: Agilent 5TC-C18, 250 × 4.6 mm; mobile phase: methanol: water: phosphoric acid = 385:115:0.14. After preparing the samples, the retention times of the sample and standard solution were compared for qualitative analysis, and the quantitative analysis was conducted by comparing the peak area ratio of the components, including lactones and acid lovastatin, to the standard lovastatin peak area.

For the detection of Ochratoxin A, the sample was thoroughly mixed, and 4.7843g was accurately weighed and placed into a 50mL volumetric flask with 95% methanol extraction solvent. The sample was sonicated for 40 minutes and extracted multiple times. The combined supernatant was concentrated under reduced pressure in a round-bottom flask to a final volume of 5mL in a graduated test tube. An appropriate amount of neutral adsorbent resin was washed three times with 95% methanol, followed by three washes with distilled water. The resin was then packed into a column (1 cm × 20 cm, 10 cm column bed height). After equilibrating the column with two bed volumes of distilled water, 1/5 of the sample concentrate was applied to the column, followed by elution with 70% methanol. The first 20 mL of eluent was collected and filtered through a 0.45 µm membrane, then ready for injection. The chromatographic conditions were as follows: flow rate 1.0 mL/min; column temperature 25°C; injection volume 20 µL; detection wavelength 238 nm; chromatographic column: Agilent 5TC-C18, 250 × 4.6 mm; mobile phase: methanol: water: phosphoric acid = 385:115:0.14. After analyzing the chromatogram obtained using a fluorescence detector, the sample was identified by comparing its retention time with that of the standard Ochratoxin A. Quantification was based on the comparison of the chromatographic peak areas between the sample and standard.

Additionally, ultra-high-performance liquid chromatography coupled with hybrid quadrupole-Orbitrap high-resolution mass spectrometry (UPLC-Q-Orbitrap HRMS) was used to profile the metabolome of the red yeast rice extract. The Thermo Scientific Accucore C18 column (100 × 3 mm, 2.6 μm) was used as the chromatographic column; acetonitrile was used as mobile phase A, and 0.1% formic acid solution as mobile phase B, with gradient elution. The flow rate was 0.3 mL/min; the injection volume was 2 µL; the column temperature was 30°C; the detection wavelength was 254 nm. The gradient elution program was as follows: 0–45 min, 25%-84% A; 45–55 min, 84%-25% A; 55–60 min, 25% A. The analysis was performed in both positive and negative ion modes using the electrospray ionization source (HESI), with full scan mode (Full-MS, m/z 100-1500 Da, resolution: 35,000) and data-dependent secondary scanning (dd-MS2, m/z 100-1500 Da, resolution: 17,500). The spray voltage was 3.5 kV (positive ion mode) and 3.0 kV (negative ion mode); sheath gas flow rate: 35 arb; auxiliary gas flow rate: 10 arb; heater temperature: 350°C; ion transfer tube temperature: 300°C; collision energy: 20, 40, 60 eV. Based on qualitative information, including retention time, exact molecular mass (m/z), and the primary and secondary MS fragmentation ions, the chemical components shared between the two red yeast fermentation products were identified in both positive and negative ion modes.
