# Supplementary figures and images for: Red yeast rice extract improves lipid metabolism by modulating gut microbiota in high-fat diet mice
Source: Front Pharmacol. 2025 Aug 1;16:1608582. doi: 10.3389/fphar.2025.1608582 (PMC12353709; doi:10.3389/fphar.2025.1608582)

Supplementary material 4: The corresponding MS/MS spectra of nine distinct compounds


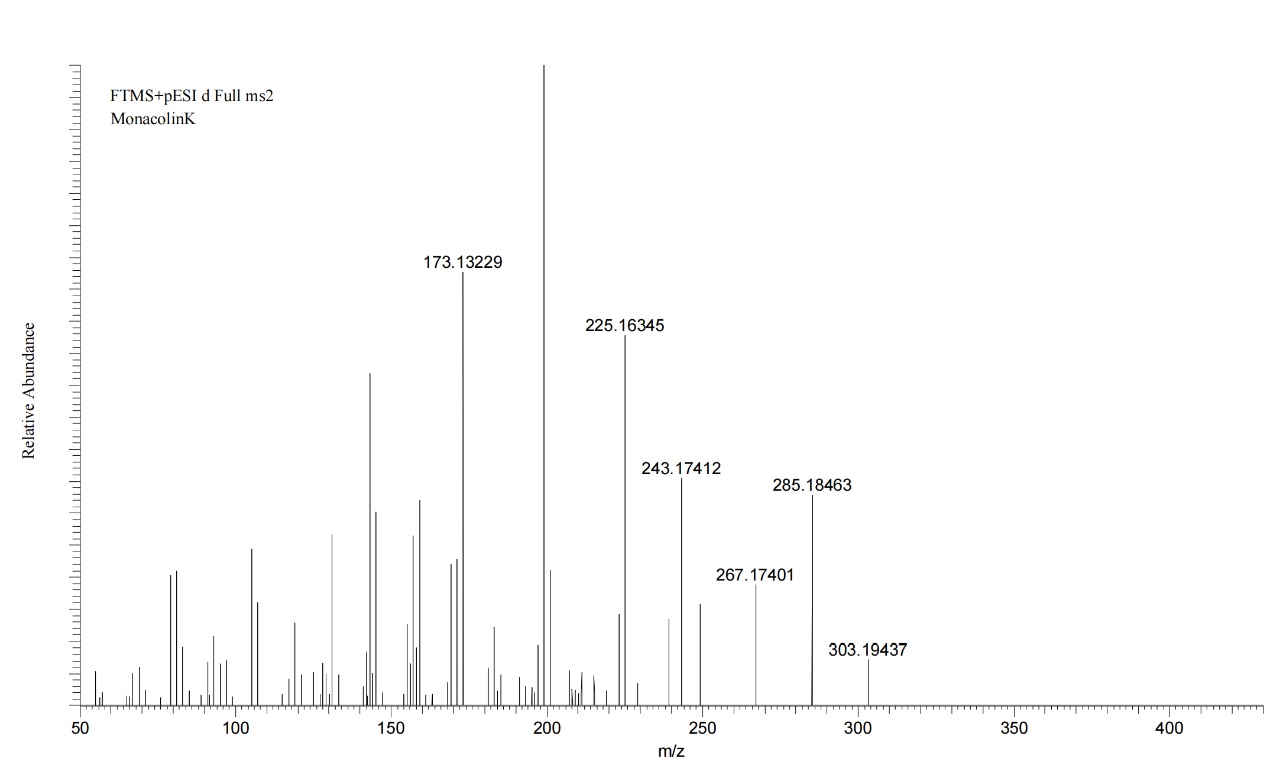


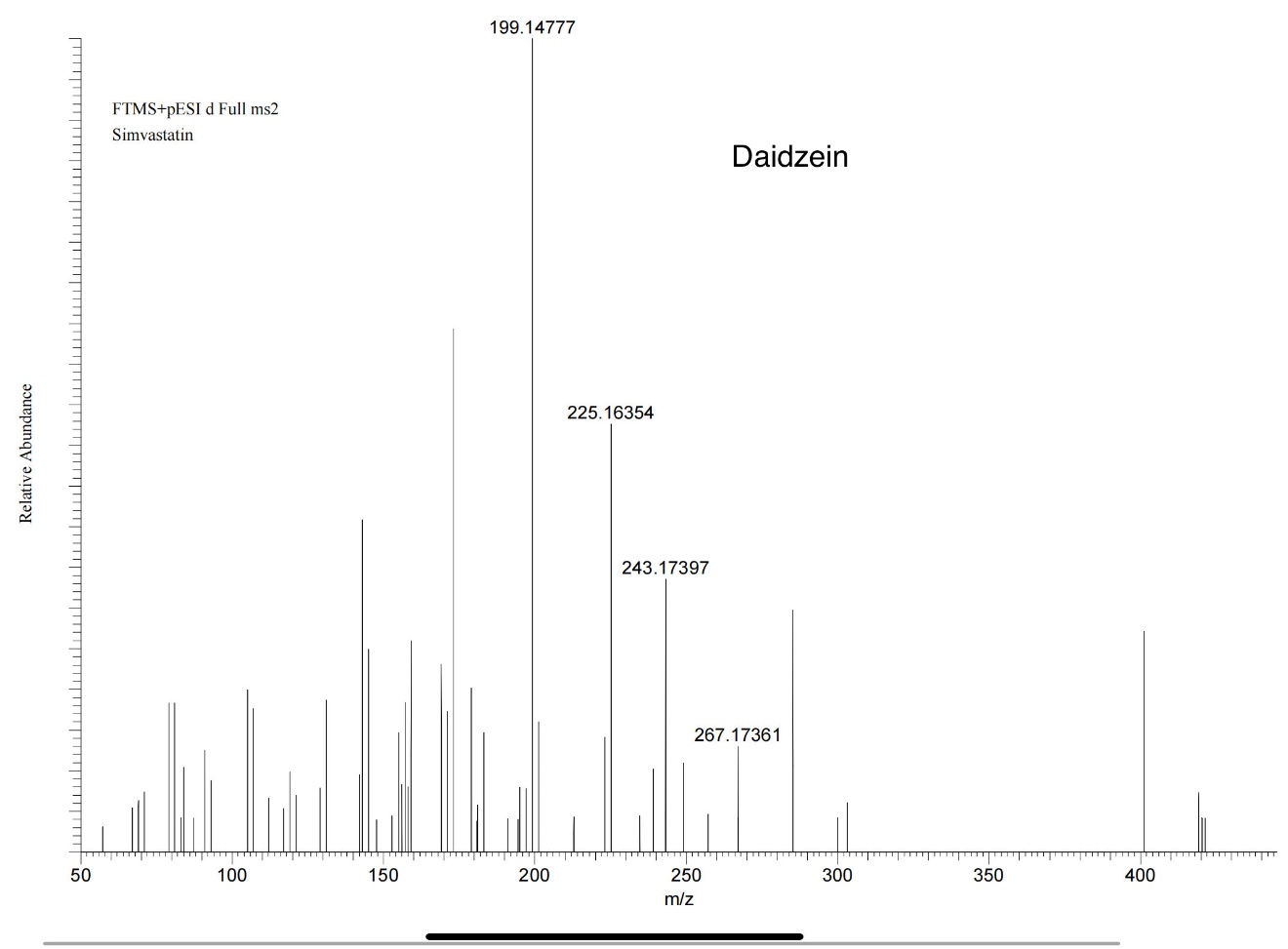


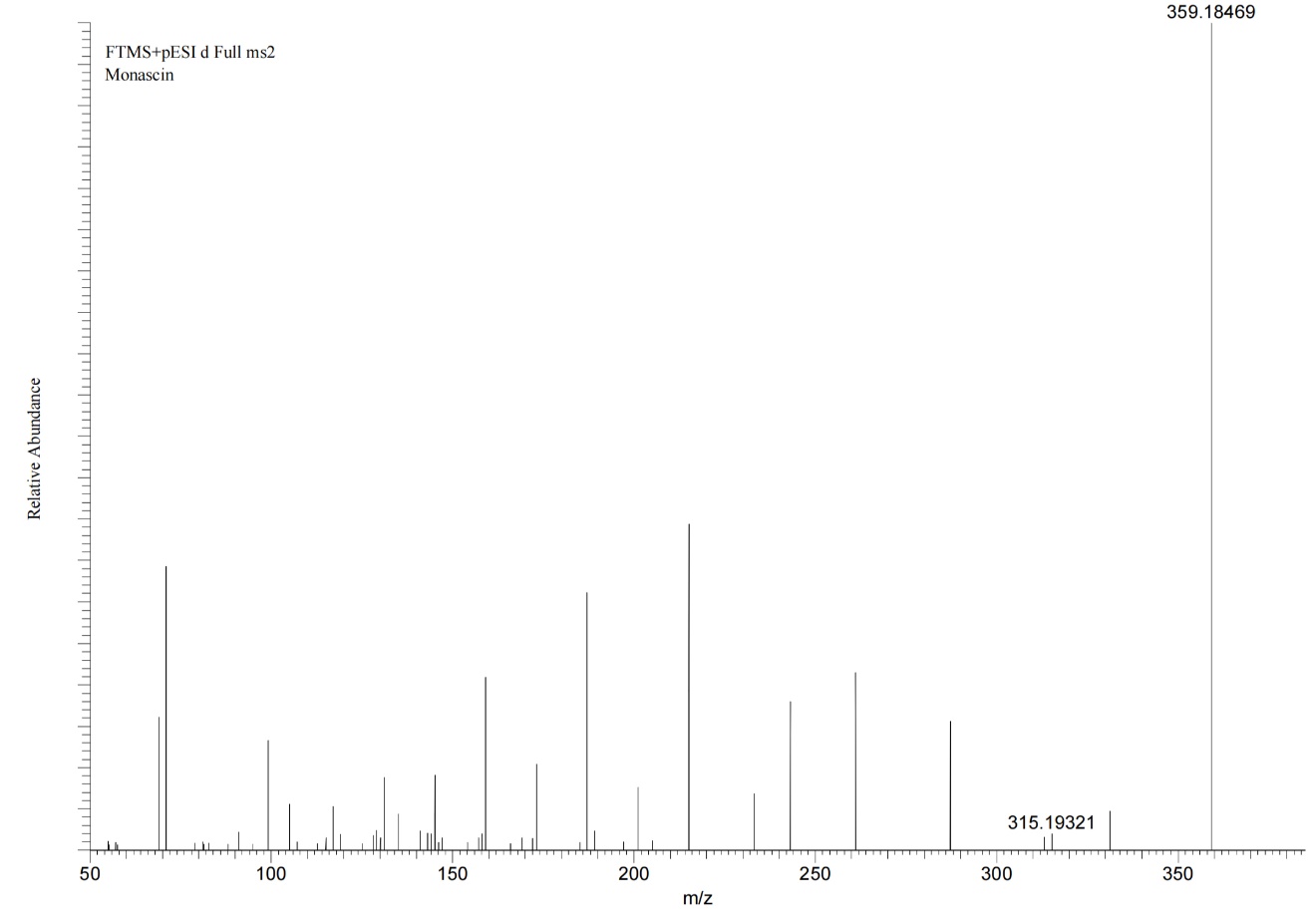


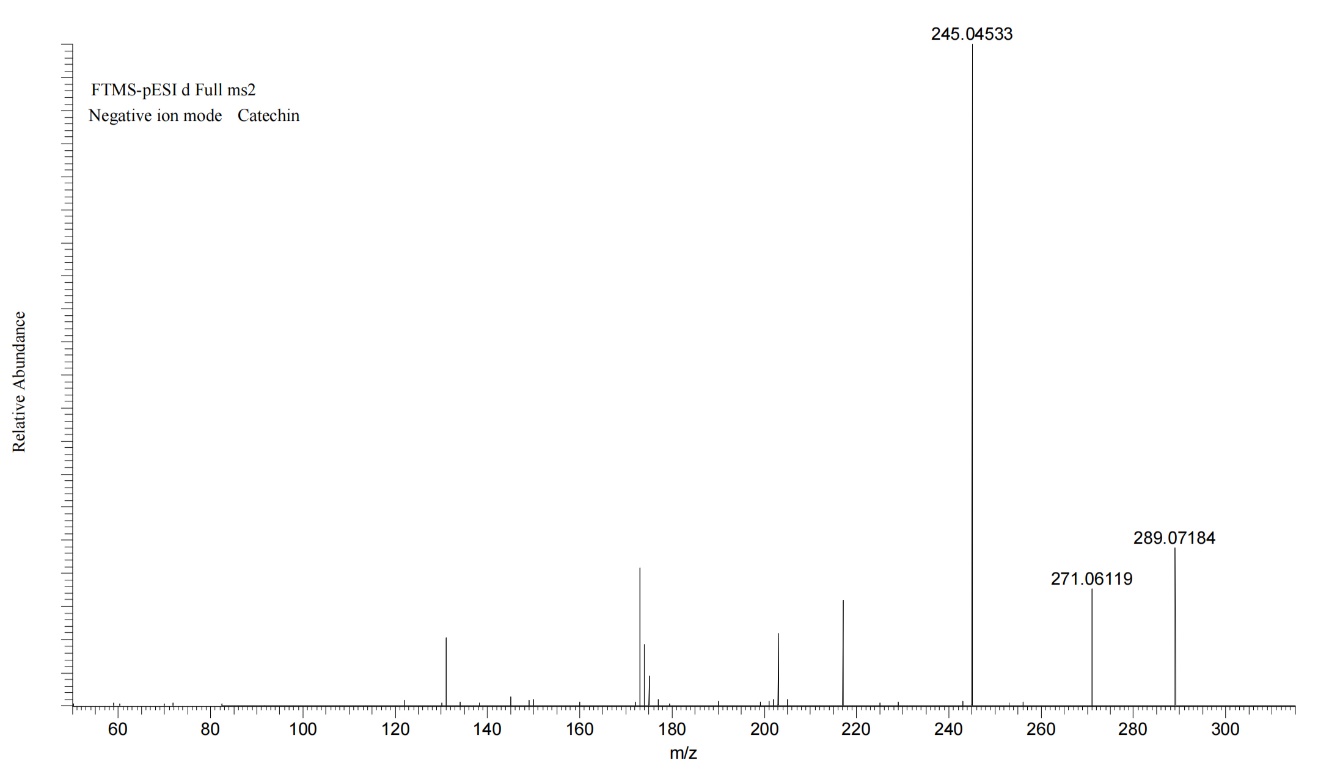

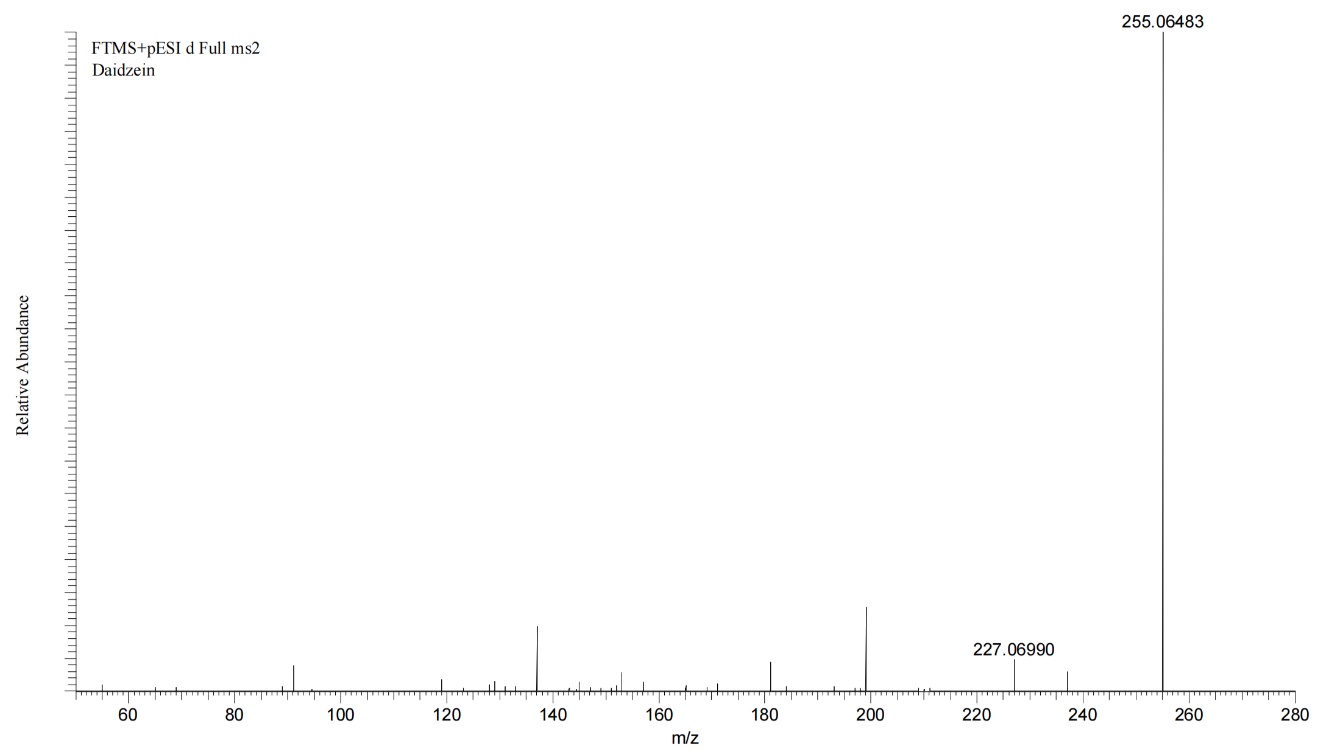


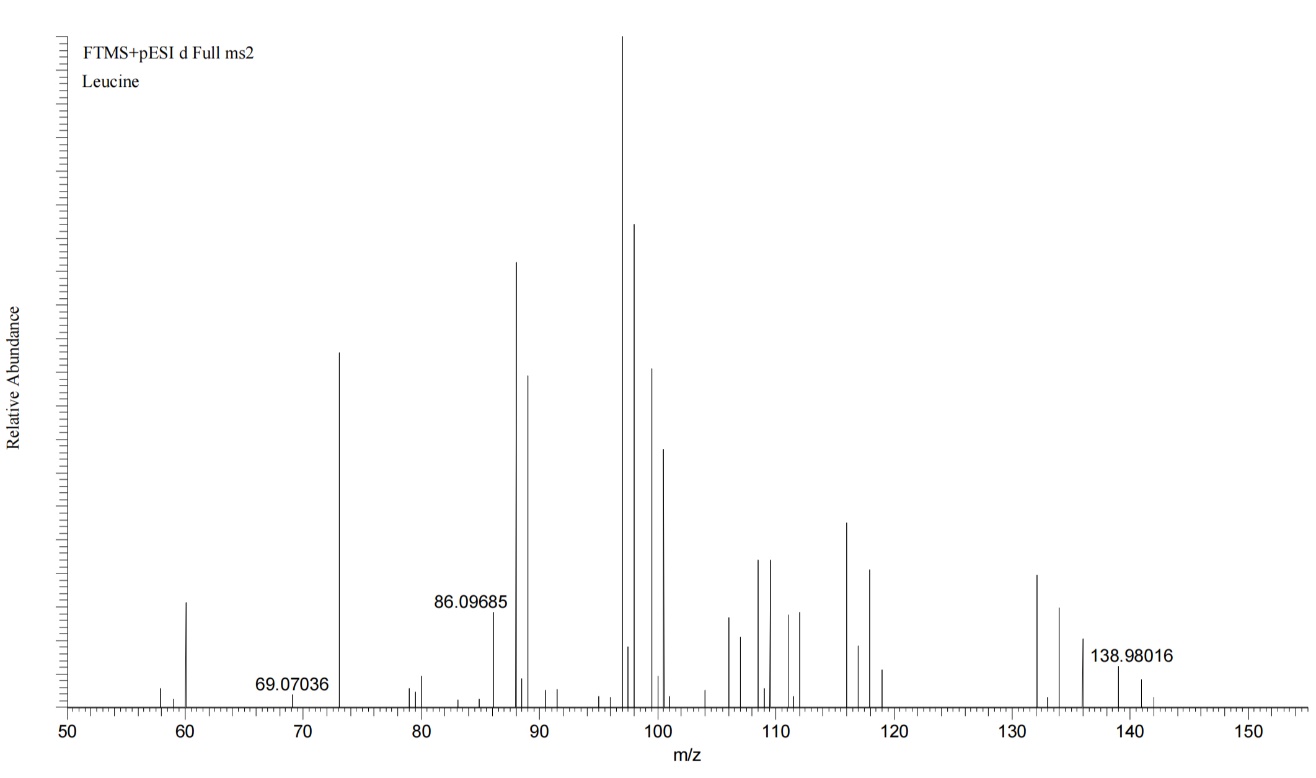

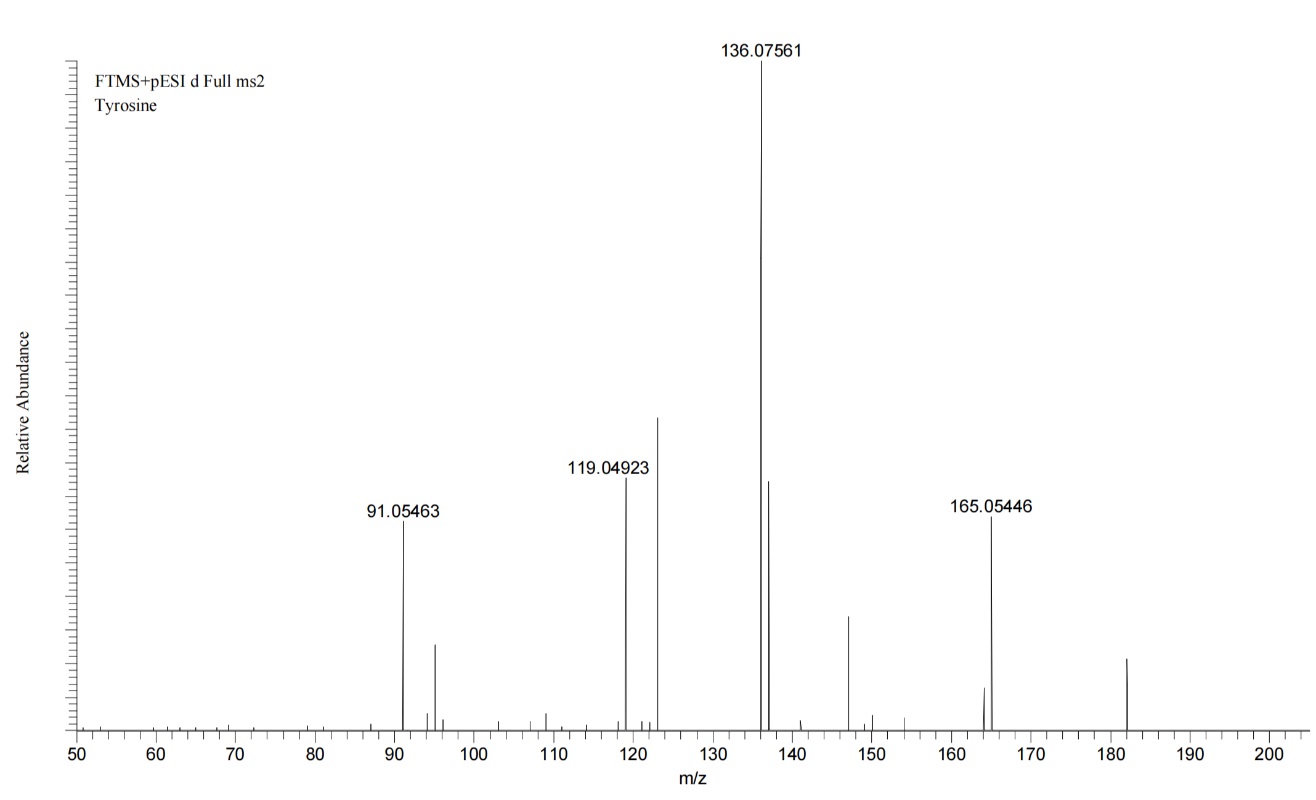


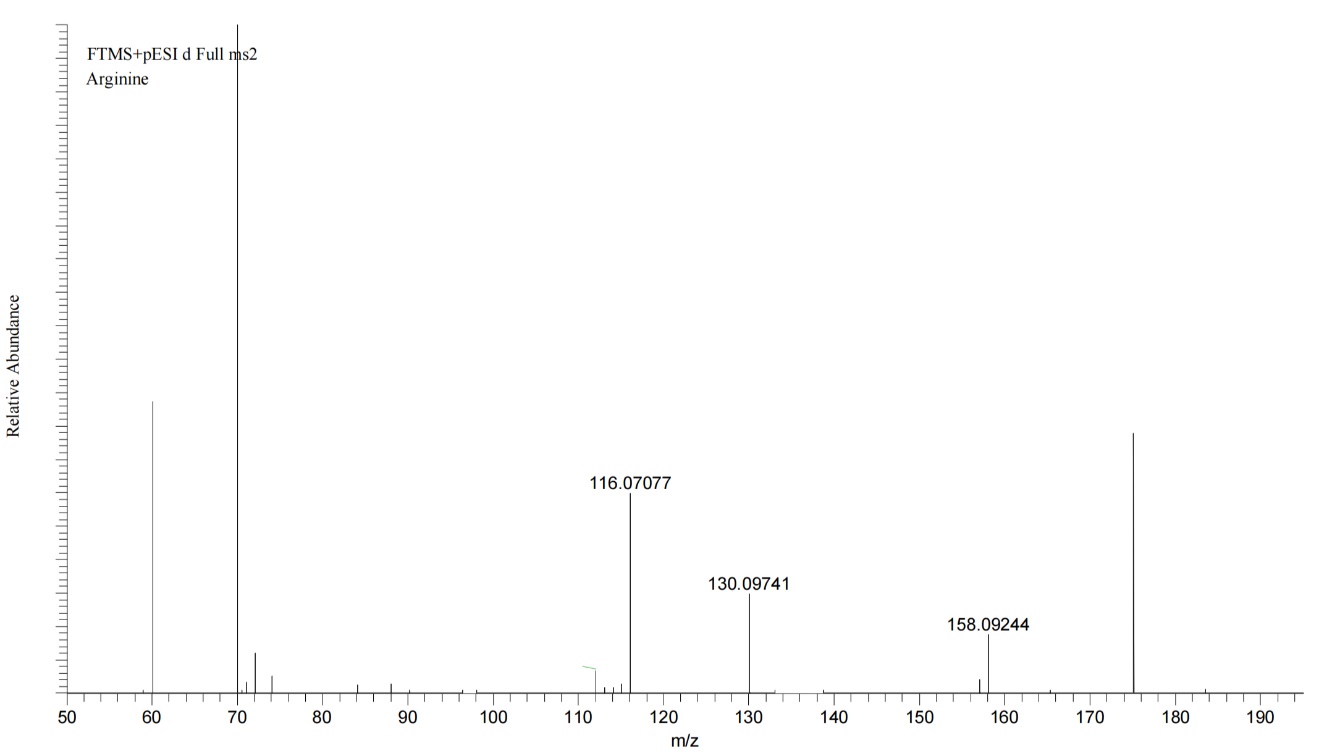

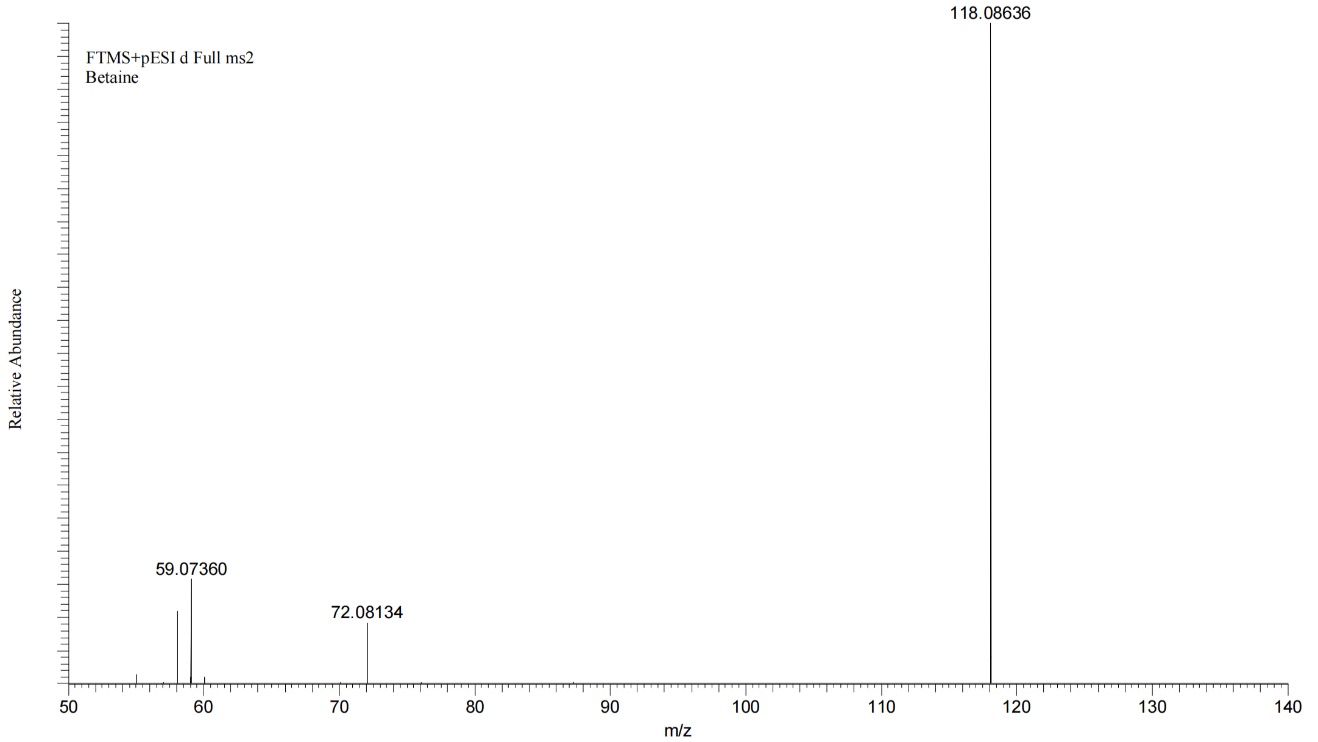

Supplement: Supplementary file 5 [file Supplementaryfile4.docx]
